# Still a Hard-to-Reach Population? Using Social Media to Recruit Latino Gay Couples for an HIV Intervention Adaptation Study

**DOI:** 10.2196/jmir.3311

**Published:** 2014-04-24

**Authors:** Omar Martinez, Elwin Wu, Andrew Z Shultz, Jonathan Capote, Javier López Rios, Theo Sandfort, Justin Manusov, Hugo Ovejero, Alex Carballo-Dieguez, Silvia Chavez Baray, Eva Moya, Jonathan López Matos, Juan J DelaCruz, Robert H Remien, Scott D Rhodes

**Affiliations:** ^1^Columbia UniversityHIV Center for Clinical and Behavioral Studies at the New York State Psychiatric Institute and Columbia UniversityNew York, NYUnited States; ^2^Columbia UniversitySchool of Social WorkNew York, NYUnited States; ^3^AID for AIDS InternationalNew York, NYUnited States; ^4^Latino Commission on AIDSNew York, NYUnited States; ^5^University of Texas at El PasoDepartment of Social WorkEl Paso, TXUnited States; ^6^The City University of New YorkCenter for HIV Educational Studies & TrainingNew York, NYUnited States; ^7^The City University of New YorkDepartment of Business and Economics - Lehman CollegeNew York, NYUnited States; ^8^Wake Forest University School of MedicineWinston-Salem, NCUnited States

**Keywords:** social media, online recruitment strategies, Spanish-speaking Latino men who have sex with men (MSM), Latino gay couples, Latino MSM, HIV prevention

## Abstract

**Background:**

Online social networking use has increased rapidly among African American and Latino men who have sex with men (MSM), making it important to understand how these technologies can be used to reach, retain, and maintain individuals in care and promote health wellness. In particular, the Internet is increasingly recognized as a platform for health communication and education. However, little is known about how primarily Spanish-speaking populations use and engage with each other through social media platforms.

**Objective:**

We aimed to recruit eligible couples for a study to adapt “Connect ‘n Unite” (an HIV prevention intervention initially created for black gay couples) for Spanish-speaking Latino gay couples living in New York City.

**Methods:**

In order to successfully design and implement an effective social media recruitment campaign to reach Spanish-speaking Latino gay couples for our ongoing “Latinos en Pareja” study, our community stakeholders and research team used McGuire’s communication/persuasion matrix. The matrix guided our research, specifically each marketing “channel”, targeted “message”, and target population or “receiver”. We developed a social media recruitment protocol and trained our research staff and stakeholders to conduct social media recruitment.

**Results:**

As a result, in just 1 month, we recruited all of our subjects (N=14 couples, that is, N=28 participants) and reached more than 35,658 participants through different channels. One of the major successes of our social media recruitment campaign was to build a strong stakeholder base that became involved early on in all aspects of the research process—from pilot study writing and development to recruitment and retention. In addition, the variety of “messages” used across different social media platforms (including Facebook, the “Latinos en Pareja” study website, Craigslist, and various smartphone applications such as Grindr, SCRUFF, and Jack’d) helped recruit Latino gay couples. We also relied on a wide range of community-based organizations across New York City to promote the study and build in the social media components.

**Conclusions:**

Our findings highlight the importance of incorporating communication technologies into the recruitment and engagement of participants in HIV interventions. Particularly, the success of our social media recruitment strategy with Spanish-speaking Latino MSM shows that this population is not particularly “hard to reach”, as it is often characterized within public health literature.

## Introduction

In the United States, online social networking features are widely used, as reflected in the Health Online 2013 survey conducted by the Pew Research Center’s Internet and American Life Project. Their report noted, for example, that more than one-third of US individuals turn to the Internet to learn about health problems [1]. Moreover, although it is difficult to gather exact figures, it is estimated that somewhere between 20 million and 40 million people in the United States visit online dating websites each month [2]. An online social network is a website or online application that allows individuals and communities to connect and communicate by sharing pictures, messages, and other forms of multimedia communication [3]. However, new media technologies are constantly emerging along with new definitions of what constitutes a social network, making it challenging to maintain a rigid definition capable of encompassing all of these rapid changes.

The rise of new communication technologies and social media in recent years has brought both new opportunities and challenges for public health professionals. Peer-to-peer exchanges through websites, online communities, and smartphone applications represent a major shift in the way people meet potential sexual partners, especially for men who have sex with men (MSM) [4-7]. Mobile accessibility through popular applications like Grindr, SCRUFF, Jack’d, and OkCupid offer new ways for MSM to engage in peer-to-peer exchanges, whether for gaining support and sharing information with fellow members of their community or to find sexual and/or romantic partners. At the same time, there has been a dramatic increase in the availability of information promoting healthy behaviors and advertising human immunodeficiency virus (HIV) and acquired immunodeficiency syndrome (AIDS) resources on the Internet, although evaluation of such strategies is often lacking [8-10].

Most of the research on social media has been focused on “risk behaviors” [4,11-15] and developing new HIV prevention and health promotion “media interventions” [16-21]. Our research responds to a key component of the research and program engagement that includes a discussion of recruitment strategies and methods for engaging subjects and participants in research and prevention programs. This particular area of research merits attention as Institutional Review Boards across the country try to comply with ethical standards and protection of participants in both online and offline contexts [22].

In particular, online social networking usage has increased rapidly among African American and Latino MSM [16,17,23], making it important to understand how these technologies could be used to reach, retain, and maintain individuals in research and medical care [24,25]. While most research on the use of social media among minorities has focused on African American and acculturated Latino MSM [26,27], little is known about those who are less acculturated, monolingual, and/or primarily Spanish-speaking Latino MSM. Interestingly, this particular group has been previously labeled as “hard to reach” by scholars and researchers [28-30]. Thus, in order to better recruit and engage this community, we assessed and implemented a recruitment campaign to enroll Latino gay couples in an HIV intervention adaptation study using both social media platforms as well as traditional approaches with community-based organizations. The goal of the study is to adapt “Connect ‘n Unite” (CNU), a couple-based intervention for stimulant-using black MSM, to reduce the disproportionate HIV burden borne by Latino MSM. CNU has been developed, implanted, and evaluated as a 4-module couple-based HIV preventive intervention. The focus of our paper will center on the need to better understand the Latino gay community, its engagement with social media, and the potential for effective delivery of wellness promotion interventions and HIV prevention programs through these media.

## Methods

### Summary

**Figure 1:**
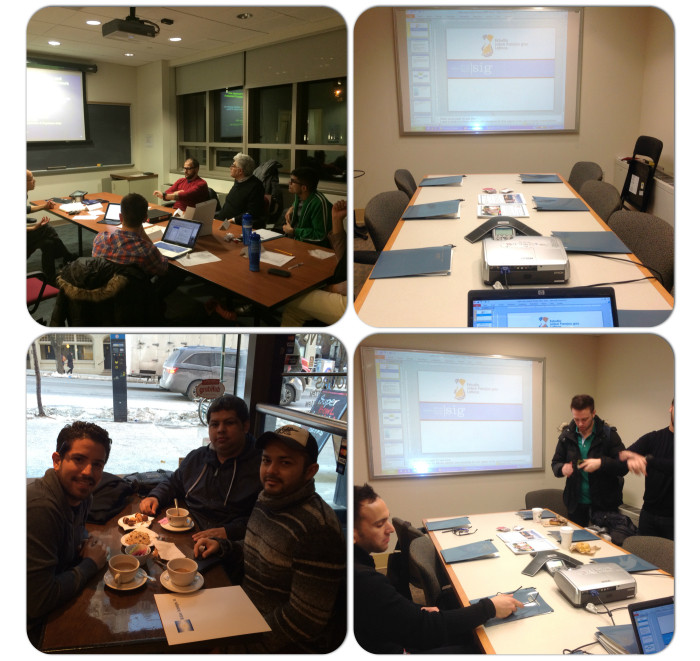
Collection of photos from a stakeholder meeting.

The Institutional Review Board at the New York State Psychiatric Institute approved this study. We undertook a mixed method approach to conduct our research. For the purpose of this paper, data analysis is restricted to the first month of the study implementation and recruitment, during which time we recruited our total sample size (N=14 couples). We relied on community stakeholders to help guide recruitment of participants through social media, from the development of materials to the wording and message of postings. A total of 12 stakeholders from a wide range of community-based organizations and Latino gay couples met weekly to develop social media recruitment materials. The principal investigator (PI) and research assistant took notes at all of the meetings (Figure 1). Stakeholders were introduced to methodological approaches in order to guide the development of social media recruitment materials. We also used McGuire’s communication/persuasion matrix as a model for understanding and creating an effective recruitment campaign [31]. Other scholars have used this model to guide evaluation of recruitment strategies for HIV prevention and wellness programs [32]. The model identifies the variables relating to effective persuasive communication, including receiver, channel, message, and source. Each of these elements is further defined and discussed below in relation to our “Latinos en Pareja” study.

### Receivers

The receiver is the intended recipient of a message. For this study, the goal was to recruit 14 Spanish-speaking Latino gay couples to participate in an intervention adaptation workshop, composed of three sessions. We aimed to adapt CNU, a couple-based intervention created for stimulant-using black MSM, to reduce the disproportionate HIV burden borne by Latino MSM. CNU has been developed, implemented, and evaluated as a 4-module couple-based HIV preventive intervention [33,34]. The modules cover several topics including self-care (eg, information about HIV/AIDS, stimulant use), communication (eg, use of effective communication styles), relationship strengthening (eg, identification of unwritten rules and sexual decision making), and couple problem solving (eg, identification support mechanism for each partner). Couples were eligible for the “Latinos en Pareja” study if they met the following criteria: (1) both partners were 18 years or older, (2) both partners considered the other male as their “main partner”, which is operationalized as (a) a male with whom he has had an ongoing sexual relationship over the prior 3 months, (b) a male considered a “boyfriend, domestic partner, spouse, ongoing lover, or regular partner”, and (c) a stated intention to remain together for at least 12 months, (3) at least one partner self-identified as Latino or Hispanic (ie, a native or inhabitant of Latin America; a person of Latin American origin living in the United States), (4) at least one partner had limited English proficiency and both partners were proficient in Spanish, (5) at least one partner reported one or more unprotected acts of anal intercourse in the past year, within or outside of the relationship, and (6) at least one partner reported using illicit substances or other drugs/substances not prescribed by a doctor that change mood or thinking in the past 3 months, or drinking more than 4 drinks in a single period or 14 drinks per week in the past 3 months. Couples were excluded if either partner reported the occurrence of one or more incidents of severe intimate partner violence within the relationship in the past year. Participants called our research telephone number to find out whether they were eligible through the phone screening.

### Channels

**Figure 2:**
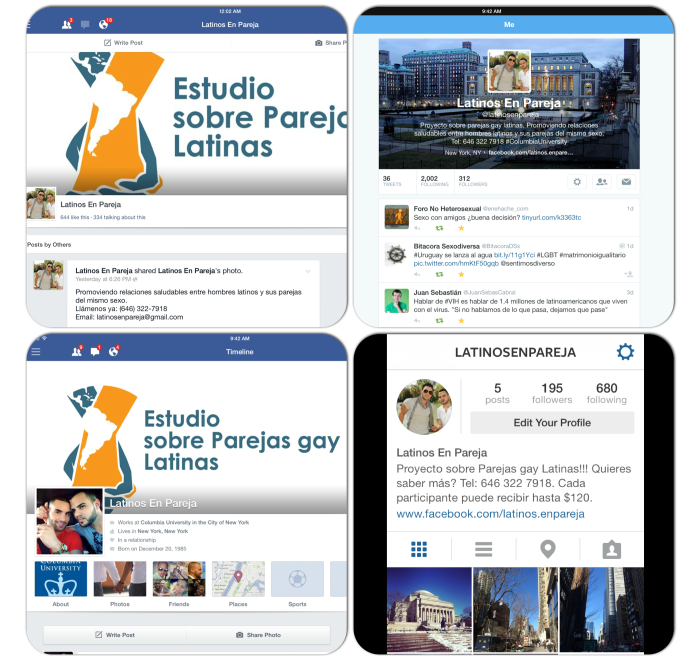
Collection of photos from our online social media sites.

Most of the participants were recruited using social media. We diversified our social media channels by reaching a wide range of social network sites, including Craigslist and Facebook as well as iPhone and other smartphone applications including Jack’d, Grindr, Twitter, Instagram, and SCRUFF (Figure 2). We created study profiles on the popular dating websites OkCupid and Adam4Adam, but site administrators quickly deleted the profiles since they promoted research. Therefore, we decided not to create new profiles on these sites even though Adam4Adam has been known as a site frequently visited by gay, bi, MSM, trans, and other sexual minority men of color. We also developed a website using Wix, a free website builder tool [35].

**Figure 3:**
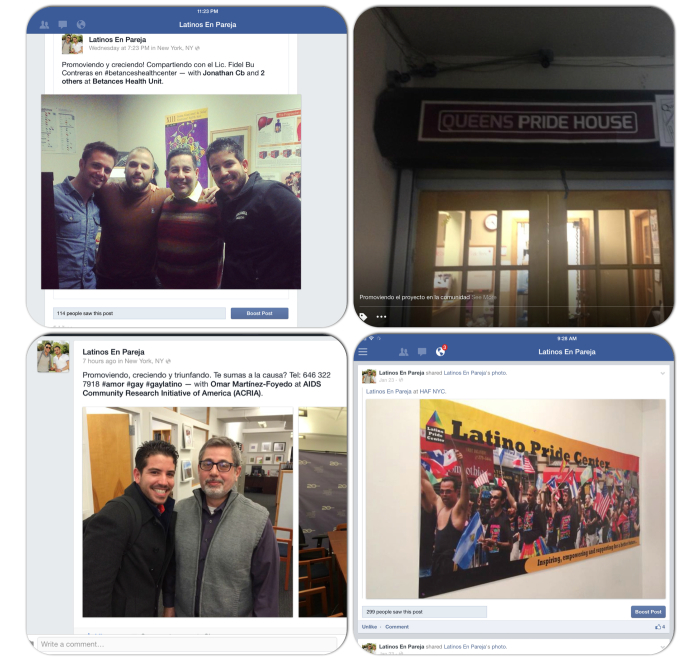
Some of the community-based venues promoted through our social media sites.

Along with recruiting participants through social media platforms, research staff who were involved in the recruitment and the adaptation of the study focused on other channels to distribute recruitment materials including local barbershops visited by gay Latinos, community-based organizations and social venues, press releases and public service announcements published through local media, and the deployment of outreach recruiters in gay venues (eg, bars, bathhouses, community events, and local support groups aimed at gay men). We also shared on our social media sites information about these venues as well as the services they offered (Figure 3). However, although we reached more than 50 community-based organizations in New York City, most of the participants came from the Hispanic AIDS Forum, Betances Health Center, Latino Commission on AIDS, and Make the Road New York.

### Messages

**Figure 4:**
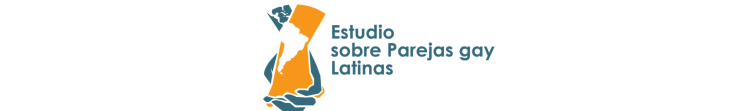
Latinos en Pareja logo.

We used a cell phone and the research center’s computer to deliver recruitment and promotional messages for the study. The message refers to the information intended to reach recipients. Stakeholder meetings, which included representatives from community-based organizations, assured the research staff that the message was culturally relevant and directed at Spanish-speaking Latino gay couples. The stakeholders discussed and offered advice on the study name and logo (Figure 4), wording of the publicity messages, visual layout of the palm cards, study’s website, and advertisements. The stakeholders, along with the research staff, suggested a very specific name, “Latinos en Pareja”, that would attract our specific target population. Furthermore, all the materials and postings with the name of the study featured a photograph of a same-sex Latino couple. A group of Latino gay couples associated with the study consented to share their pictures for advertising and promotional purposes related to the study.

**Figure 5:**
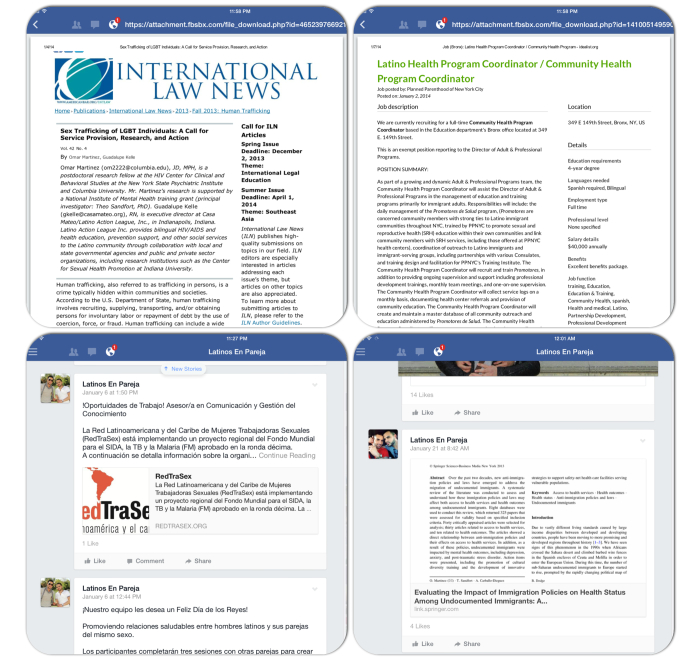
Collection of photos with job opportunities and information on social issues.

Developing the study’s recruitment message for social media channels such as Facebook and the study website also included describing the program as “Promoviendo relaciones saludables entre hombres Latinos y sus parejas del mismo sexo [Promoting strong healthy relationships between Latino men and their same-sex partners]”. Stakeholders also recommended including language that would conjure up images of building something for the good of the collective, “Los participantes completarán tres sesiones con otras parejas para crear programas innovadores de prevención del VIH en nuestra comunidad [Participants will complete three sessions with other couples to help build stronger HIV prevention programs in our community]”. Additionally, stakeholders recommended that we include postings related to social causes such as same-sex marriage and job and volunteer opportunities (Figure 5). Stakeholders also stressed the importance of including photographs showing the diversity of the Latino gay community, in terms of age, country of origin, race, socioeconomic status, gender, body type, and sexual preferences. Potential participants who had expressed interest in the study and “friended” our pages had the opportunity to respond to these postings and share their couples stories, anecdotes, and photos on our sites.

We used a more simplistic approach in publicizing our message through the smartphone applications such as Grindr, SCRUFF, and Jack’d. Our profile heading included the words “En pareja [In a relationship?]?” and a picture of a couple holding each other. The couple was part of our stakeholder team who consented in writing to use of the picture. Once potential participants clicked on the profile, they were directed to the following heading, “Proyecto sobre Latinos en Pareja [Latino gay couples study]”, and a brief description that included the following message, “Proyecto sobre latinos en pareja. Promoviendo relaciones saludables. Cada participante puede recibir hasta $120. Nos puede llamar ahora para determinar si es elegible [Latino gay couples study. Promoting healthy relationships. Each participant can receive up to $120. Call us to determine if you are eligible]”, plus a phone number and email address. Participants also had the option to access our Latinos en Pareja Facebook page, using the social network link built into the apps.

### Source

Our research study was run from two prominent research centers at Columbia University: the HIV Center for Clinical and Behavioral Studies and the Social Intervention Group (SIG). In particular, we received recruitment support, guidance, and training by the HIV Center Media Core through various opportunities, including Cross-Core Meetings, which are built to provide guidance for new studies at the HIV Center. In addition, the study received the support from research staff at the Wake Forest University School of Medicine, North Carolina, and the Department of Social Work at the University of Texas at El Paso.

## Results

### Ethical Considerations in Conducting Social Media Recruitment

The use of social media for recruitment of participants involves a number of new ethical considerations that must be integrated into the training of stakeholders and research staff and into study protocols to ensure proper protection of potential participants. Prior to recruitment of participants, stakeholders and research staff met to outline strategies to protect potential participants’ confidentiality and to discuss proper ways to approach potential participants. Stakeholders recommended the development of a protocol, which was later developed by a collaborative team of stakeholders and research staff (Multimedia Appendix 1). In addition, research staff and stakeholders received training on how to recruit and engage participants through social media for the purposes of cultivating interest in the study.

Regarding Facebook recruitment, we agreed that the Facebook group and study page should have the same name as the study, “Latinos en Pareja”, and must be open to the public so it could be found by open searches and members could invite others to join. Importantly, all group members could post public messages, notes and pictures, and engage in private messages and chats. Facebook users were made aware that their postings, including photos, may be used for the purposes of the study via a disclaimer on our Facebook page. In addition, all of the photos published in this manuscript were shared directly with the research team by Facebook users to use for study-related deliverables. The PI monitored the Facebook group and page and responded to private messages and inquiries about the study with the approved New York State Psychiatric Institute Institutional Review Board (NYSPI IRB) language. Research staff had weekly discussions with stakeholders to ensure privacy was upheld in all of our social media channels. We did not collect identifying data for any of the potential participants who contacted us for information about the study. However, as approved by the NYPSI IRB, those who screened eligible were given the option to provide their personal information in order to be contacted for the study. All identifying data for the study comply with the Health Insurance Portability and Accountability Act (HIPAA) regulations, and storage of data is encrypted, firewalled, and password-protected. Although more ethical issues will emerge as online engagement continues, we have sought to address major ethical concerns both in the training and study methods.

### Social Media

We relied on a wide range of social media and community-based venues for recruitment (Table 1). Potential participants were instructed to call our direct research line to be screened to determine their eligibility. Facebook proved to be the most successful tool to recruit participants into the study. By the end of January 2014, a month after the study was initiated, and after having successfully recruited all the eligible couples, we had 1872 members associated with the Facebook individual profile and 1370 members in the Facebook group. We reached approximately 6612 weekly through the Facebook interactive page with a total of 589 friends. Additionally, statistical analytical tools built into the Facebook group page enabled us to keep track of the “likes” and the number of individuals “reached” on a weekly basis. A total of 44 participants were screened through Facebook, and 5 of the 14 eligible couples were recruited using this site. “Liking” other causes or groups enabled “Latinos en Pareja” to share targeted recruitment materials on the pages of other groups and causes. For instance, the Hispanic AIDS Awareness Program, a nationally well-known advocacy organization, shared several of our postings to help promote the Latino gay couples study. In return, we also shared and endorsed their site on our page. Our Facebook group particularly targeted other groups catering to Latinos on topics such as Latino parties in the Jackson Heights neighborhood of Queens, New York, wellness promotion events, and social venues for Latino gay men. By the end of the first month of the study, we had hundreds of members associated with our Facebook page. Some of these partner groups on Facebook include “Gay men interracial lovers”, “Gay Latinos in NYC and Friends”, “New York Latinos Underground”, “Gay Latinos Worldwide”, and “Core-Group Latinos D”.

**Table 1:** Summary of recruitment reach through social media channels and community-based venues.

Social media sites (Jan. 1-31, 2014)		Number of potential participants reached or total number of messages (N=35,658)	Eligible couples (N=14)
**Facebook**			5
	Screeners	44	
	Messages	1000	
	Individual profiles	1872	
	Groups	1370	
	Pages	589	
	Total number of posts	120	
	Weekly page reach	6612	
**Grindr**			0
	Screeners	21	
	Messages	300	
**SCRUFF**			0
	Screeners	1	
	Messages	200	
**Jack’d**			0
	Screeners	5	
	Messages	250	
**Craigslist**			0
	Screeners	4	
	Postings	10	
	Messages	50	
**Twitter**			
	Screeners	0	0
	Following on Twitter	2003	
	Followers	315	
	Tweets	36	
	Messages	150	
**Instagram**			0
	Screeners	0	
	Following on Instagram	667	
	Followers	167	
	Posts	5	
**Community-based organizations**			5
	Hispanic AIDS forum Screeners	4	
	Betances	3	
	Latino Commission on AIDS Screeners	2	
	BOOM! Health Screeners	2	
	Make the Road New York Screeners	2	
**Others**			4
	Screeners through couples or friends’ referral	18	

**Figure 6:**
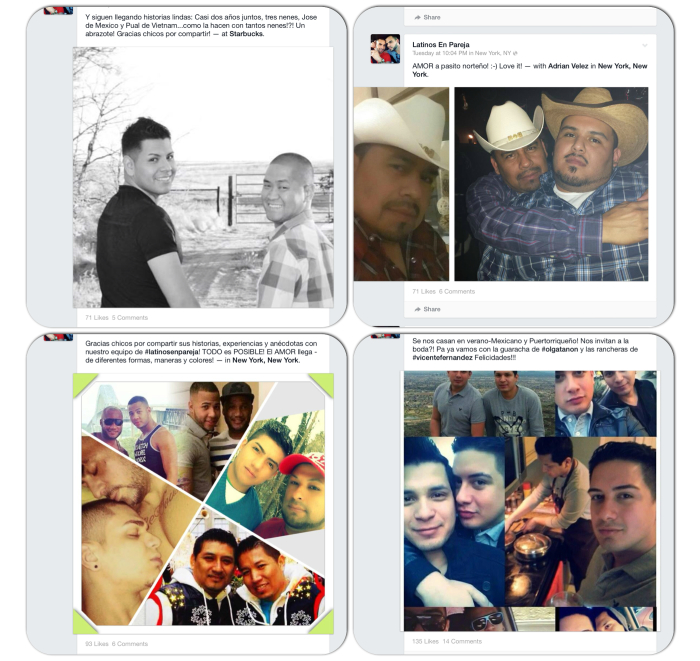
Collection of photos shared directly with the research team by Facebook users with their consent to use for study-related deliverables.

Many potential participants saw our Facebook profile as a reflection of the value of collectivism in Latino communities as well as a networking tool enabling them to share their personal stories about being a Latino gay couple. Interestingly, many potential participants also visited the profile as single Latino gay men hoping to find other partners. In addition, members shared events and parties taking place in the community. In particular, members found merit in our strategy to post jobs and volunteer opportunities in the New York City area on the group page. Stakeholders used the group page to refer jobs to member, and we also used Indeed, the popular job search engine, to advertise job and volunteer opportunities to group members. Many couples also shared their picture, stories, anecdotes, and provided advice and suggestions on how to maintain healthy relationships (Figure 6).

Our Instagram application enabled us to share pictures related to couples and HIV prevention and to send messages that Latino gay couples would identify with using the popular social media tool of hashtags, which allows social media users to create trends, share threads of information, and reach a wider general audience. Some of our hashtag messages included #latinosenpareja, #*Prevención*delVIH, #amordeparejas, #amorenpareja, #justicia, #*acción, among many others*. The Instagram application was shared and connected with our Facebook page, helping us streamline and integrate all of our communications and messages. Twitter was also used to shared news and important events affecting the Latino MSM community. The same strategies were used on Twitter by joining relevant profiles sharing similar interests and causes and targeting the same group of Latino MSM.

Our website featured recruitment information about the study, pictures of couples, positive messages including our objective in “promoviendo relaciones saludables [promoting healthy relationships]” and “crear programas innovadores de prevención del VIH en nuestra comunidad [creating innovative HIV prevention programs in our community]”, as well as information about stakeholders, volunteers, and the research team. In addition, through the website, we constructed a “Contact Us” inbox where potential participants could email us with their inquiries about the study as well as other related questions [35].

These built-in tools in our channels, including Internet pages and smartphone applications, enabled us to increase our engagement through social media. In total, we received 3670 messages from social media sites, screened 75 potential participants of the total 106 screened, and recruited 7 eligible couples through social media. Most of our screeners on smartphone applications were from Grindr, with a total of 21 potential participants screened.

Engaging with various social media platforms led to an interactive dialogue between potential participants, community stakeholders, and the research team. In particular, our social media presence and messages received a great deal of positive feedback. Individuals engaged with our online social media tools shared their thoughts, pictures, and opinions on the study through a public forum that connected them to a community of like-minded individuals. For example, one individual stated on our Facebook wall:

Estoy promoviendo este espectacular proyecto de investigación que la Universidad de Columbia y un grupo de amigos están desarrollando en la ciudad de Nueva York. Si te consideras un hombre comprometido con tu pareja y responsable con tu entorno, no deberías quedar ajeno a esta iniciativa que pretende cambiar el modo en que enfrentamos el mundo. Además, si somos amigos es porque te considero alguien valioso y quiero lo mejor para ti. Por eso te invito a que junto a quien amas vivan esta experiencia. [I’m promoting this wonderful research study developed by Columbia University and a group of my friends in New York City. If you are a man in a committed relationship with your partner and responsible in your community, you should not miss out on this new initiative that is trying to change the way we deal with the problems we face in our world. Furthermore, if we are friends, it is because I consider you as someone who is valuable and I want the best for you. For this reason I’m inviting you and your loved one to join us in this experience.]

Facebook user

Another Facebook user shared “Gracias por su presencia. Existe falta de difusión de forma que la ayuda llegue más proporcionalmente. Ustedes son un buen ejemplo de ayuda en información que se necesita casi urgente. Saludos [Thank you for your presence. There is a real need to reach out and spread the word more efficiently. You are a good example of a source of useful information that is urgently needed]”

Furthermore, social media proved to be an effective tool for sharing HIV information and knowledge, testing resources, and agency referrals. Potential participants discussed and responded to our information with thoughtful insights about factors leading to HIV and barriers to medical care. One Facebook user explained:

Cabe destacar que el incremento del HIV tiene mucho que ver con tres factores muy importantes: 1) economía 2) educación y 3) ambiente. Aunque todos estamos expuestos a una variedad de enfermedades de transmisión sexual, pobre y ricos, las personas de bajos recursos económicos son más propensas al contagio. También existen factores como la promiscuidad y comportamiento relacionado al consumo de alcohol y drogas ilícitas. Por otra parte, la educación sexual escolar es mínima o no existe en los planteles escolares públicos, lo que contribuye a la intimidad sexual sin conocer los riesgos que implica falta de higiene y protección con anti-conceptivos u otros métodos conocidos como condón o abstinencia. Y por último, personas que viven en áreas pobres o de bajos recursos económicos, zonas urbanas o suburbios, tienden a vivir sin el cuidado de personas adultas. Por otro lado, vivir en confinamiento, apartamentos u hogares de menor espacio en relación a la cantidad de personas residiendo bajo un mismo techo, también se puede considerar como un grave problema que aumenta la posibilidad de adquirir tanto HIV como otras enfermedades relacionadas al contacto sexual. [It is worth mentioning that the rise in HIV has to do with three important factors: (1) the economy, (2) education, and (3) the environment. Although all of us are exposed to a variety of sexually transmitted diseases, both rich and poor, people with fewer economic resources are more prone to infection. There are also other factors to consider such as promiscuity and behaviors related to the consumption of alochol and illegal drugs. On the other hand, sexual education in schools is very minimal or does not exist in public schools, a fact that contributes to sexual intimacy without knowing the risks that implies a lack of hygiene or protection using contraceptives or other known methods like condoms and abstinence. And finally, people who live in poor areas or those with fewer economic resources, in urban or suburban zones, tend to live without the supervision of adults. On the other hand, living in confined spaces, in cramped apartments or homes with many people under the same roof, also could be considered a serious problem that increases the possibility of acquiring HIV and sexually transmitted infections.]

Our focused and targeted message about the study and the participants’ positive perception of our study enabled us to increase recruitment and engage others through all of the social media venues we used. In addition, through the sharing of sexual health information, we enabled users to engage in thoughtful discussions about HIV, sexually transmitted infections (STIs), and sexual health promotion.

### Community-Based Organizations and Other Recruitment Venues

We reached more than 50 community-based organizations in the five boroughs of New York City (Bronx, Brooklyn, Manhattan, Queens, and Staten Island). However, a large majority of the potential participants screened through community-based organizations came to us from Hispanic AIDS Forum (n=4), Betances Health Center (n=3), Latino Commission on AIDS (n=2), BOOM! Health Center (n=2), and Make the Road New York (n=2) (Table 1). In total, we recruited 5 eligible couples for the study through community-based organizations. In addition, 4 eligible couples were enrolled through referrals from other couples or friends. We also recruited in barbershops where most of the clientele were Latino and lesbian, gay, bisexual, and transgender (LGBT) individuals, including Pride Cuts in Manhattan.

## Discussion

### Principal Findings

Our findings show that monolingual Spanish-speaking Latino MSM who are less acculturated create strong and diverse networks through social media. This engagement goes beyond “joining a group” and expands into online social ties that in turn translate into social integration, informational support, communication, and engagement. This could be explained by the idea that Latino immigrants, vis-à-vis their US-born counterparts, form strong social ties and social support groups [36-38]. Our initial findings on participants’ engagement with social media platforms associated with the study suggest that the value of collectivism that is part of Latino communities carries over into online communities targeting Latinos as well. In addition, our findings underscore the importance of social media ties and interactions in the development of programs and interventions to improve health outcomes. Individuals were comfortable discussing topics related to couples, including sexual health, non-monogamous relationships, disclosure of HIV status, and communication skills, among others. Individuals also shared their thoughts and the need to build couple-based programs and counseling services.

We also encourage and suggest that social media networking sites fully endorse and support research institutions in their endeavors to address complex health challenges affecting vulnerable populations. In particular, social media should help promote low-budget studies that are not able to spend on major advertisement campaigns through their sites. We do understand that this commitment comes with mutual respect and agreements. Social media sites and mobile applications, including Grindr, SCRUFF, and others, might benefit from the established networks as a result of the program or intervention. For instance, social media partners could engage those established networks by the research team through other channels including community-based organizations and Facebook. As the influence of social media expands, social media sites owe and share in the social responsibility to promote community health, and thus they should actively engage with researchers, social activists, and community stakeholders.

Another important topic to consider is the sustainability of the study sites and built networks. The PI will maintain the website as a resource channel for Latino gay couples well after the completion of the research funding cycle. This site could serve as potential space to house future recruitment initiatives for other studies targeting Latino gay couples. In case funding alternatives do not work for couple-based studies, the PI will train community stakeholders to manage the site and will encourage monthly meetings to engage in discussion and continue supporting those involved in the site. Other social media channels, such as Grindr, SCRUFF, and Jack’d, will be deactivated at the conclusion of the recruitment phase of the study.

### Limitations

Given the limited resources available to evaluate the success of our social media recruitment tools to deliver information, a detailed qualitative and quantitative content analysis of the social medial channels was not feasible. In order to maintain high ethical standards using new social medial tools, we refrained from disclosing personal messages sent by participants that could have provided further insights in regards to the enhancement of participant engagement and recruitment. In addition, we could not access the impact of delivered information and shared knowledge on participants, including the effect of the shared HIV and STI information, as well as referral references and how these might have impacted participants’ behaviors. Future studies should further analyze how Spanish-speaking Latino MSM might benefit from a built-in online HIV prevention intervention using existing channels such as Facebook, Grindr, and SCRUFF, to mention a few.

### Strengths

Our success relied on the ongoing support from our stakeholders and community partners who were involved throughout the recruitment stage by providing critical feedback, refining messages and contents, and guiding the process of recruitment. As other studies have shown, the involvement of stakeholders in the research process is key to the success of programs, interventions, and research [39-42]. Our research staff benefited from this type of partnership and collaboration, and it will hopefully extend into the implementation of the HIV prevention intervention for Latino gay couples at the community level at a later stage of the process. Stakeholders also benefited from this collaboration. During the meetings, the research staff provided access to and discussed up-to-date research on the use of social media and technology to disseminate health information and promote wellness. In addition, three community stakeholders were introduced, connected, and enrolled in free English summer courses with the English as a Second Language (Community Impact Program) Program at Columbia University. Community involvement extends to all aspects of our adaptation research process and will continue expanding by building trust and exchanging information, sustaining relationships and commitments, and sustaining and maintaining knowledge, capacity, and values generated from the partnership.

### Conclusions

Our findings provide initial support for the feasibility of recruiting a diverse group of monolingual Spanish-speaking Latino gay couples and notes the strategies, including targeted messaging, needed to recruit this population—a population that we have found to not be so “hard to reach” after all if social media platforms and community outreach efforts are combined in a strategic and culturally appropriate way. The study’s social media presence reached a substantial number of fans and continued to engage community members and serve as a platform for interacting and sharing information for Latino MSM. Our interactive approach to developing recruitment materials, implementing recruitment strategies, and filtering content feedback with community stakeholders allowed ongoing improvements to the recruitment materials. In the end, this interactive approach expanded our reach within communities of gay and other Latino MSM using these important social media network spaces. The use of multiple social media proved highly effective to provide a window into a population that is often characterized as “hard to reach”.

## Multimedia Appendix 1

Social media protocol.

## Abbreviations

AIDS: acquired immunodeficiency syndrome
CNU: Connect ‘n Unite
HIPAA: Health Insurance Portability and Accountability Act
HIV: human immunodeficiency virus
LGBT: lesbian, gay, bisexual, and transgender
MSM: men who have sex with men
NYSPI IRB: New York State Psychiatric Institute Institutional Review Board
PI: principal investigator
STI: sexually transmitted infection
