# Supplementary material for: Still a Hard-to-Reach Population? Using Social Media to Recruit Latino Gay Couples for an HIV Intervention Adaptation Study
Source: J Med Internet Res. 2014 Apr 24;16(4):e113. doi: 10.2196/jmir.3311 (PMC4019772; doi:10.2196/jmir.3311)
Supplement: Supplementary file 1 [file jmir_v16i4e113_app1.pdf]

Latinos En Pareja: Social Media Outreach Protocol

I) Protocol for Social Media Outreach Implementation

Internet Outreach - Any type of engagement with potential participants via social media, email, smart phone application, chat rooms and/or other web based listings about the "Latinos en Pareja" (LEP) project.

- **i.** Staff will conduct outreach on social media sites that are welcoming to Latino MSM.
- **ii.** Staff will be looking at social media, chat rooms, smart phone applications and networking sites that are LGBT friendly, including: Grindr, Scruff, Jack'd, Adam4Adam.com, Facebook, Twitter, Instagram and Craigslist are examples of suitable internet venues for internet outreach.
  1. Any other sites should always be approved by the principal investigator before staff posts any recruitment material or other project related language is posted to a site.
  2. Staff will post advertisement slogans and/or recruitment posters on sites and areas that are likely to attract participants. Any material to be posted must first be approved by the Principal Investigator.

II) How to Conduct Outreach

a. Internet Outreach

- **i.** When staff is conducting Internet outreach in any chat room site, smart phone application, or social media page, a basic profile should be created in order to ensure the maximum number of participants can be recruited from the information listed on the profile page.
- **ii.** When creating a basic LEP profile page or account name for a chat room, staff should consider the terms of services for that particular website. Staff should have approval from the Principal Investigator before a profile is created.
- **iii.** A basic LEP profile, which includes relevant information about the project, and a LEP password will be created for login as the overall login for any site used for Internet outreach or other engagement with potential participants through these venues.
- **iv.** All encounters and information from talking, chatting, and/or instant messaging to participants should be recorded after session is completed (See participant database -- located under the Outreach tab in the LEP Project Google doc).
- **v.** The username and password will be available for use by any staff member and any type of social media outreach to include, but not limited to, Facebook, Twitter, Instagram, etc. These accounts should be monitored by

staff at least 2-3 times a day to address all questions from possible participants surrounding interest in the project.

b. Community-based outreach combined with internet outreach

i. If in person, hand him a study flyer, promote our social media sites (e.g., Facebook, Grindr, scruff), and describe the project).

ii. We are a team of researchers at the NYSPI and Columbia University and community partners conducting a study on Latino men and their same-sex partners (18 or older) in the New York City area. Latino men and their same-sex partners will complete three sessions with other couples to help build stronger HIV prevention programs in the community. Each session last approximately 2 hours. Both partners must participate. Each participant can receive up to $120.We think this is an exciting, new project because it is specifically for Latino gay couples!
Is this something you feel you can and would like to be part of? Yes or No

IF NO TO Q1: Okay, I am sorry to hear that. Do you mind telling me why? Your reason may help us plan future groups. [Record Reason for Refusal Below. After recording their reason, say:] Thank you for your time today. If you change your mind, please call us at the number provided on the back or you can contact us through our social media channels (e.g., Facebook, Grindr, study website). Also, if you know other men or male couples who might like to and be able to participate before *[ANTICIPATED RECRUITMENT CLOSE DATE]*, feel free to give them the flyer with our contact information.
Reason(s) for Refusal:

____

IF YES TO Q1: Great! Proceed to screening. Please make sure there is privacy. I will need to ask you some personal questions to make sure that you are eligible to participate. Some questions may seem silly and obvious, but I still need to ask them. Some of these questions include the use of drugs and alcohol, information about your partner, HIV status, sexual behavior, and intimate partner violence. If you are eligible, I will take your information, so that we can follow-up with you and __*[PARTNER'S FIRST NAME OR PSEUDO NAME]*__ to schedule the first session.

If no privacy, ask individual for contact information, time availability, and name or pseudo name to follow up with him and proceed with screening.

III) Consent Form Waiver

a. People will have voluntarily call the principal investigator or research staff to screen for study participation after seeing flyers, information from social media channels, have heard about the study by word-of-mouth, or have voluntarily given their contact information to staff during recruitment outreach. The screening will take 20 minutes at most, and the participant has the direct opportunity to refuse to participate in the study immediately after screening; of course the participant can decline participation at any point during the screener or the study. Passive declines are respected in that after three attempts to contact a person for screening, they are withdrawn from the active list and their information is destroyed. After

screening is completed and participants are deemed eligible, they will be told more about the process they will encounter when they arrive for their scheduled sessions, including information about written informed consent, time involved, and reimbursement for their time and travel expenses.

IV) Recording of Outreach Information

a. Staff should follow this protocol for all types of outreach and engagement when recruiting participants for the project.

i. Any contact information will be recorded in the LEP participant database (located in the LEP Project Google doc). All necessary information should be obtained to record the most accurate information for contact so that a participant can be scheduled for screening.

ii. After an outreach engagement, staff will go to the participant database in the LEP Project Google doc and will enter requested information:

1. Name
2. Date
3. Time
4. Type of Contact (Grindr, Scruff, Jack'd, Facebook, Twitter, etc.)

iii. Staff should follow this process for all types of Internet outreach, included but not limited to the sites mentioned above. If engagement is made through a chat room venue or a social media site the staff member who is engaging with the potential participant should record all information as asked in the LEP participant database and record that information into that database electronically and follow-up the next day so that the participant can be contacted about doing a screening.

1. Facebook/Adam4Adam/e-mail/other chat rooms: When a participant makes contact with LEP, the following response should be sent: "Thank you for contacting Latinos En Pareja. My name is (xxx), and I am excited that you are interested in our project. In order for me to get you the right information, can you please provide me with an email address or phone number where I can contact you? If you feel comfortable, may I also have your first name?" After this stock message is sent, all communication with this potential participant should be conducted via the Latinos En Pareja email address (Username: latinosenpareja@gmail.com password: _________________)
    a. All accounts created for outreach should be checked at least once a day by a research assistant (See above re: how to initially engage participant).
2. All initial contact information (and date of contact) should be recorded in the participant database.

A research assistant will then contact potential participants listed in the participant database (See initial contact script for more information). Once a participant indicates that he is interested in LEP, he should be scheduled for a screening using the Latinos En Pareja shared calendar (Note: staff may access this calendar through the above Gmail account). Please contact the Principal Investigator for any questions regarding scheduling a participant for screening.

V) Internet Emergency Situations

a. Response to emergency situations

i. Do not leave the site and try to engage the participant.
ii. Call the Principal Investigator. Inform him of what is happening, and actions taken.
iii. If a participant is ill or injured call 911 for medical attention.
iv. If a participant poses an imminent threat to the safety of himself or herself or to other participants, call 911 for law enforcement intervention.
v. The principal investigator or research staff must ensure that appropriate mental health or health services are provided to participants as needed.

VI) Internet Averse Event

a. Description of an adverse event

i. Events leading to serious psychological, social or physical harm to participant
ii. Events that result from his participant in the study, including responding to assessment interviews or being exposed to intervention activities.
iii. Events that are reported to or observed by any study staff.

b. Examples of adverse events

i. Acute stress, concern, emotional upset or agitation related to taking part in an interview. For example, participants might be affected by talking about sensitive matters, HIV and risks they may have taken.
ii. Violations of confidentiality or privacy. An example is having information about their participation disclosed by staff.
iii. Harassment or violence. Examples are violence directed at a participant as a result of taking part in the screening or some study activity; physical or verbal abuse from a partner as a result of participating in a study activity.
iv. Complaints about inappropriate or unprofessional behavior on the part of any staff member.
v. Severity of an adverse event: Mild – an event that is considered a problem for a few hours. Moderate – an event that is considered a problem for a few days. Severe- an event that is considered a problem for weeks or months.

c. Response to adverse events

i. Adverse events must be taken seriously and handled in a professional and consistent manner by all research areas.
ii. All adverse events must be discussed with the principal investigator and his suggestions must be recorded and followed.
iii. Events and any action taken must be carefully recorded using the Adverse Event Report Form.
iv. A copy of any adverse event must be:
    a. Kept in participant file
    b. Submitted to principal investigator (Note: if the event occurs during online recruitment or a session include a copy of the online communication or session audio tape with report).

VII) Intimate Partner Violence (IPV) Referral

a. Educate yourself about intimate partner violence – Read attached guide and know what services are available in the community.

i. Let go of any expectations you have that there is a “quick fix” to intimate partner violence or to the obstacles a victim faces. Understand that “inaction” may very well be the participant’s best safety strategy at any given time.

ii. Believe the participant and let the participant know that you do.

iii. Listen to what the participant tells you. If you actively listen, ask clarifying questions, and avoid making judgments and giving advice, you will most likely learn directly from the participant what it is he needs.

iv. Validate the participant's feelings. It is common for abused individuals to have conflicting feelings – love and fear, guilt and anger, hope and sadness. Let the participant know that her feelings are normal.

v. Avoid victim blaming. Tell the participant that the abuse is not his fault. Reinforce that the abuse is his partner's problem and his responsibility, but refrain from "bad-mouthing" him.

vi. Take participant's fears seriously -- If you are concerned about the participant's safety, express your concern without judgment by simply saying, "Your situation sounds dangerous and I'm concerned about your safety".

b. If participant indicates that the partner has perpetrated severe physical IPV (i.e. punching, kicking, slamming against the wall, beating up, burning or scalding, choking or using a gun on them), or rape, you need to indicate in a calm, nonjudgmental way that you are very concerned about his safety because of "x, y, z" Summarize reasons.

c. All sites, online shared files (google doc), and project facilities should have a comprehensive up-to-date referral manual with Domestic Violence Programs that have a range of services, including a 24 hour crisis hotline number, legal services, shelter, counseling for women, men and children affected by Domestic Violence, as well as housing and job placement services.

d. When making referral, the principal investigator and assistant research scientist should know what are eligibility restrictions on services (i.e. are there geographic catchment area, income or insurance issues that would prohibit participants from receiving services), and if possible, have a contact name and phone number of someone at the referral source, who will be responsive to following through with the referral.

VIII) Confidentiality

a. It is crucial that team members protect the confidentiality of study participants and the confidentiality of participants in other SIG studies. Please refer to Columbia's RASCAL training website for more information on confidentiality (https://www.rascal.columbia.edu/).

IX) Process to resolve conflict

a. If you experience a problem with a co-worker, your first suggested action is to speak directly with that co-worker. If you feel uncomfortable doing so, or if working directly with the stakeholder does not resolve the issue, please see the Assistant Research Scientist to resolve any conflicts or concerns that you may have. If your conflict is with the Assistant Research Scientist, please try to resolve the matter between the both of you. However, if that fails or if you do not feel comfortable doing so, please see the Principal Investigator in an effort to resolve the conflict (Note: Omar is supervising Zach, so he should be contacted if there is a need to mediate a conflict with Zach).

b. We very much encourage open and direct communication to work through conflicts. We do not recommend discussing co-worker problems with other SIG staff, as this often leads to counterproductive communication that exacerbates

conflict. We really want to create a strong working team and a positive work environment where everyone feels supported in doing their work.

c. Please see the SIG Resource Manual for additional staff related protocols.

X) Safety Measures for Outreach

a. Staff should always have a cellular device when conducting outreach in order to effectively communicate/ask any general comments/questions to staff or the Principal Investigator.
b. For immediate emergencies any staff conducting outreach should contact the Columbia University Department of Public Safety numbers for the following emergencies.
c. To reach the Department of Public Safety for a security, fire or medical emergency dial:
    i. Morningside - On campus- 99 / Off campus- 212-854-5555
    ii. Medical Center - On campus-212-305-7979 / Off campus- 212-305-8100
    iii. To contact the New York City Police/Fire Department or Ambulance service dial: 911
    iv. If staff is conducting social media outreach after hours outside of their home, the staff member should notify the Principal Investigator at least several hours prior if possible to give awareness that the staff member(s) will be out after hours. Duration of outreach activity and location of outreach activity information should be given to the Principal Investigator.

Note: Monthly outreach deliverables may be found in the LEP Project Google docs.
